# Plasmonic Optoelectronic Memristor Enabling Fully Light‐Modulated Synaptic Plasticity for Neuromorphic Vision

**DOI:** 10.1002/advs.202104632

**Published:** 2021-12-29

**Authors:** Xuanyu Shan, Chenyi Zhao, Xinnong Wang, Zhongqiang Wang, Shencheng Fu, Ya Lin, Tao Zeng, Xiaoning Zhao, Haiyang Xu, Xintong Zhang, Yichun Liu

**Affiliations:** ^1^ Center for Advanced Optoelectronic Functional Materials Research Key Laboratory for UV Light‐Emitting Materials and Technology (Northeast Normal University) Ministry of Education 5268 Renmin Street Changchun 130024 China

**Keywords:** Ag—TiO_2_ nanocomposite, fully light‐modulated synaptic plasticity, localized surface plasmon resonance (LSPR), neuromorphic vision, plasmonic optoelectronic memristors

## Abstract

Exploration of optoelectronic memristors with the capability to combine sensing and processing functions is required to promote development of efficient neuromorphic vision. In this work, the authors develop a plasmonic optoelectronic memristor that relies on the effects of localized surface plasmon resonance (LSPR) and optical excitation in an Ag–TiO_2_ nanocomposite film. Fully light‐induced synaptic plasticity (e.g., potentiation and depression) under visible and ultraviolet light stimulations is demonstrated, which enables the functional combination of visual sensing and low‐level image pre‐processing (including contrast enhancement and noise reduction) in a single device. Furthermore, the light‐gated and electrically‐driven synaptic plasticity can be performed in the same device, in which the spike‐timing‐dependent plasticity (STDP) learning functions can be reversibly modulated by visible and ultraviolet light illuminations. Thereby, the high‐level image processing function, i.e., image recognition, can also be performed in this memristor, whose recognition rate and accuracy are obviously enhanced as a result of image pre‐processing and light‐gated STDP enhancement. Experimental analysis shows that the memristive switching mechanism under optical stimulation can be attributed to the oxidation/reduction of Ag nanoparticles due to the effects of LSPR and optical excitation. The authors' work proposes a new type of plasmonic optoelectronic memristor with fully light‐modulated capability that may promote the future development of efficient neuromorphic vision.

## Graphical abstract

A novel plasmonic optoelectronic memristor is demonstrated for the first time relying on localized surface plasmon resonance (LSPR) effect. Both fully light‐modulated and light‐gated electrically‐driven synaptic modulation can be implemented in such a single device. Furthermore, combination of visual sensing, low‐level (contrast enhancement and noise reduction), and high‐level image processing (image recognition) promotes the development of efficient neuromorphic vision.

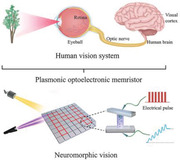

## 1 Introduction

Neuromorphic vision has attracted major interest because of its ability to emulate human visual perception, which provides specific advantages over conventional machine vision.^[^ ^1^, ^2^, ^3^, ^4^ ^]^ A machine vision system usually consists of three separate units, comprising the image sensors (photodetectors), the memory, and the processing unit.^[^ ^5^, ^6^, ^7^, ^8^, ^9^, ^10^ ^]^ However, the lack of neuromorphic functions and the physical separation of the sensing and processing parts of this system would generate large quantities of redundant data and high power consumption.^[^ ^11^, ^12^ ^]^ In contrast, the human visual system (HVS) possesses the capability of combining the sensing and processing functions: the retina can simultaneously perform the sensing and low‐level image processing such as noise suppression, filtering, and feature enhancement, thus minimizing the transfer of redundant data; the visual cortex then functions to perform parallel high‐level image processing such as recognition, classification, and localization.^[^ ^1^, ^2^, ^13^, ^14^, ^15^, ^16^, ^17^ ^]^ Therefore, inspired by the HVS, the concept of near‐/in‐sensor computing architecture is developed by Chai et al. guiding the way toward high‐efficiency neuromorphic vision, while the neuromorphic nanodevices with capability of functional combination of sensing and processing are the critical foundation of its hardware implementation.^[^ ^16^, ^17^ ^]^

Previous advances indicate that optoelectronic memristors or neuromorphic devices offer promising platforms for emulation of human visual functions.^[^ ^17^, ^18^, ^19^ ^]^ In particular, realization of a retina‐like capability to combine optical sensing and low‐level pre‐processing functions has been reported in several typical works in the field.^[^ ^10^, ^20^, ^21^, ^22^ ^]^ For instance, Zhou et al. developed a MoO*_x_*‐based optoelectronic memristor that can effectively improve the processing efficiency and image recognition rate.^[^ ^10^ ^]^ Sun et al. demonstrated the in‐sensor reservoir computing via 2D SnS memristors, prompting the development of optoelectronic memristors for language learning.^[^ ^17^ ^]^ Light‐induced synaptic potentiation or depression functions have been demonstrated in various materials, including CeO*_x_*, MoO*_x_*, Nb:SrTiO_3_, nanocrystalline Si, 2D MoS_2_, and SnS, in which the mechanisms were generally attributed to light‐induced electron trapping, valence changes, persistent photoconductivity, or enhancement of the ion migration barrier.^[^ ^10^, ^17^, ^19^, ^23^, ^24^, ^25^, ^26^ ^]^ However, the reversible synaptic modulation (depression or potentiation) behavior of these materials must be controlled using electrical stimulation, which generates complex operations involving hybrid optical/electrical signals.^[^ ^23^, ^25^, ^26^, ^27^ ^]^ To date, very few works have demonstrated all‐optical control of the conductance in this type of device.^[^ ^21^, ^28^ ^]^ The mechanism involved can be regarded as an extension of the existing models, that is, light stimuli at different wavelengths can induce the trapping/detrapping of the electrons or an increase/reduction in the number of trapping centers.^[^ ^24^, ^29^, ^30^ ^]^ However, the available physical models and materials for fully light‐controlled optoelectronic memristors remain very limited. Therefore, the exploration of novel optoelectronic memristors through introduction of a specific photoresponsive effect represents cutting‐edge research.

The localized surface plasmon resonance (LSPR) of nanostructured metals is an attractive plasmonic phenomenon that offers many advantages in terms of photoresponsivity, including wavelength tunability, a real‐time detection capability, and a rapid response.^[^ ^31^, ^32^, ^33^ ^]^ These merits indicate that it would be interesting to introduce LSPR into memristive devices, thereby enabling development of a new type of optoelectronic memristor. The problem with this idea is that the LSPR generally presents a transient photoresponse; however, an optoelectronic memristor usually requires not only the photoresponse capability but also a persistent conductance change *ΔG* induced by light irradiation.^[^ ^34^, ^35^ ^]^ Importantly, Ohko et al. proposed a photochromic nanocomposite composed of a TiO_2_ film loaded with Ag nanoparticles (NPs) that can change its color reversibly with persistent properties under both ultraviolet (UV) light and visible (vis) light.^[^ ^36^ ^]^ In our previous work, we also developed a photochromic Ag/TiO_2_ film for application to long‐term holographic data storage.^[^ ^37^, ^38^, ^39^, ^40^ ^]^ Inspired by these advances, the Ag—TiO_2_ nanocomposite provides an excellent platform for construction of an LSPR‐based optoelectronic memristor with a full light modulation capability. However, to the best of our knowledge, the related research has not been demonstrated experimentally to date.

Here, we demonstrate a plasmonic optoelectronic memristor based on the LSPR effect experimentally for the first time. In this device, a nanocomposite composed of Ag nanoparticles loaded into a TiO_2_ nanoporous film is sandwiched by Au and fluorine‐doped tin oxide (FTO) electrodes. Fully light‐induced and light‐gated synaptic plasticity functions are achieved in this single device, including the reversible synaptic potentiation/depression and the modulation of STDP learning rule under UV/vis illumination. This device is capable to perform the functional combination of visual sensing, low‐level image pre‐processing (contrast enhancement and noise reduction), and high‐level image processing (image recognition). The multifunctional photoelectric device proposed here provides a feasible route toward construction of a highly efficient neuromorphic vision system.

## 2 Results and Discussion

**Figure 1:**
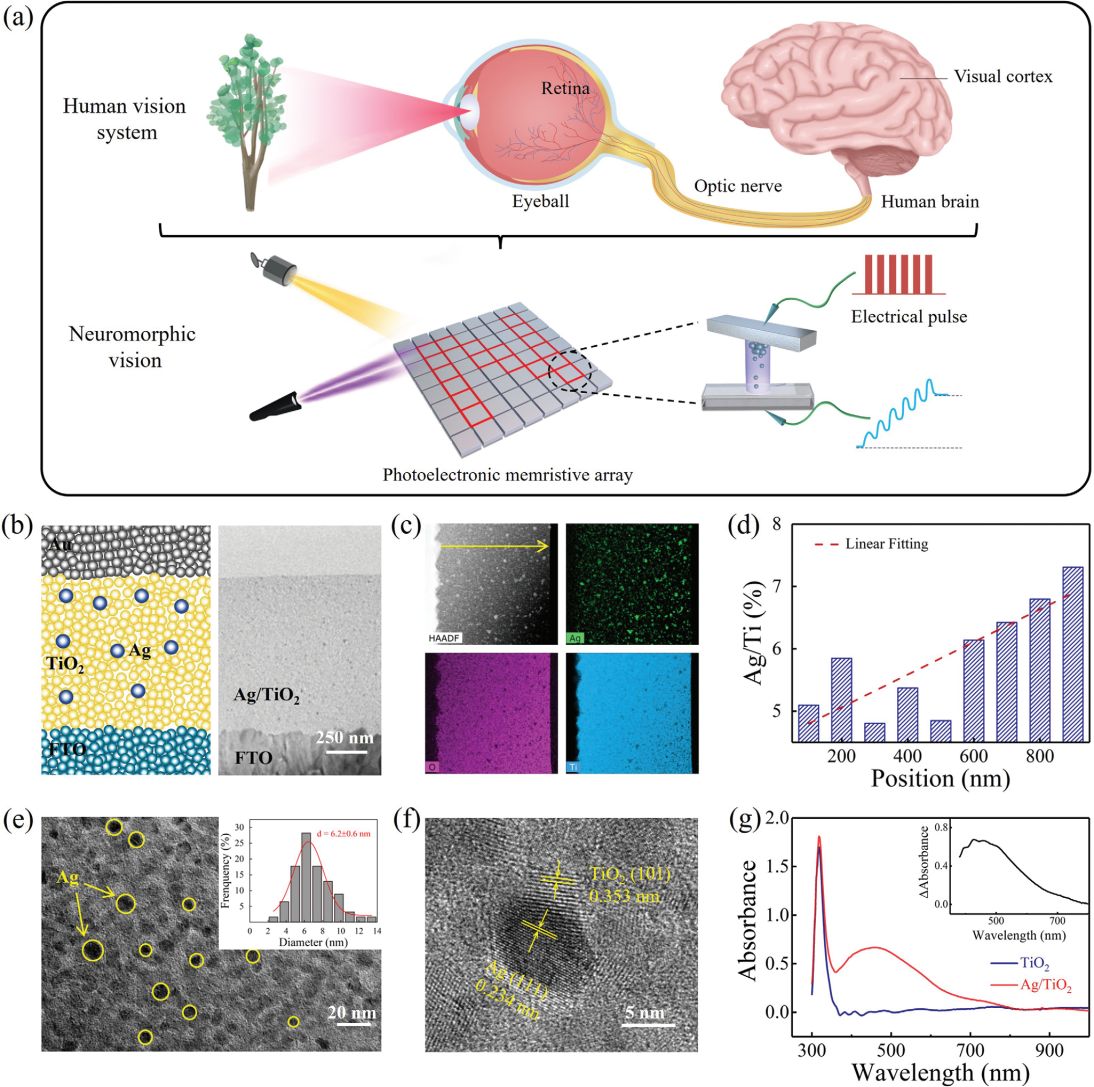
Plasmonic optoelectronic memristor with multifunction capability for neuromorphic vision. a) Schematic diagrams of human vision system and neuromorphic vision using the optoelectronic memristor. b) Device structure and cross‐sectional TEM image of the Ag—TiO_2_ nanocomposite‐based memristor. c) High‐angle annular dark‐field imaging and elemental mapping images of the Ag—TiO_2_ film. d) EDX scan analysis results for the Ag—TiO_2_ film along the horizontal arrow indicated in part (c). e,f) HRTEM images of the Ag/TiO_2_ heterostructure. The inset in (e) shows the statistical size distribution of the Ag NPs. g) Absorption spectra of the Ag—TiO_2_ film and a pure TiO_2_ film. The inset shows the differential spectrum.

**Figure** **1**a shows a schematic diagram of the human vision system and illustrates the motivation for design of an optoelectronic memristor with the capability of functional combination of sensing and processing. As the main components of the human visual system, the retina and the visual cortex are naturally responsible for a variety of visual functions. The retina of the eyeball first captures the image information and converts it into electrical signals. Importantly, the retina also performs the low‐level pre‐processing required for information compression. These signals are then transmitted to the visual cortex through the optic nerve. Finally, the visual cortex functions by performing parallel high‐level processing and memorization of the image information acquired.^[^ ^20^, ^41^ ^]^ To emulate the human visual system closely, we propose a plasmonic optoelectronic memristor that combines the visual sensing and image processing functionalities.

Figure 1b shows a schematic illustration of our proposed plasmonic optoelectronic memristor, which consists of an Au/Ag‐TiO_2_/FTO sandwich structure. The Ag—TiO_2_ nanocomposite film in this structure is prepared by photocatalytic loading of Ag nanoparticles into a nanoporous TiO_2_ film (see the Experimental section; Figure S1, Supporting Information).^[^ ^37^, ^38^, ^39^ ^]^ The right panel of Figure 1b shows a cross‐sectional transmission electron microscopy (TEM) image of our device that confirms that the uniform Ag—TiO_2_ film thickness is ≈1 µm. Corresponding energy‐dispersive X‐ray (EDX) and mapping spectra indicate that the Ag element is distributed throughout the TiO_2_ matrix with an atomic percentage of 2.33% (Figure 1c). The EDX scan analysis was conducted along the yellow line shown in Figure 1c and the Ag concentration gradient shown in Figure 1d illustrates that the atomic ratio of Ag/Ti clearly increases from the bottom to the top of the Ag—TiO_2_ film. This gradient may be caused by attenuation of the UV light in the porous TiO_2_ film during the Ag reduction process. The high‐resolution TEM (HRTEM) images in Figure 1e,f further confirm that the Ag nanoparticles are distributed randomly within the TiO_2_ film and have an average size of 6 nm. The lattice fringes at 0.234 and 0.353 nm correspond to the (111) plane of Ag and the (101) plane of anatase TiO_2_, respectively.^[^ ^42^, ^43^ ^]^ Figure 1g shows the absorption spectra of the TiO_2_ film and the Ag—TiO_2_ film. Using the TiO_2_ film as a reference, steep absorption is observed at wavelengths smaller than 390 nm, corresponding to the TiO_2_ bandgap (3.2 eV), but the absorption in the visible region is negligible. In contrast, the Ag—TiO_2_ film presents intense absorption characteristics in the visible region (400–800 nm) composed of two bands centered at ≈400 and ≈500 nm, as confirmed by the differential absorption spectrum (see the inset of Figure 1h). According to the literature,^[^ ^36^, ^37^, ^38^, ^39^ ^]^ the appearance of these absorption bands in the visible region can be attributed to the LSPR absorption property of the Ag nanoparticle, which proves the introduction of LSPR effect in our Au/Ag—TiO_2_/FTO memristor device.

**Figure 2:**
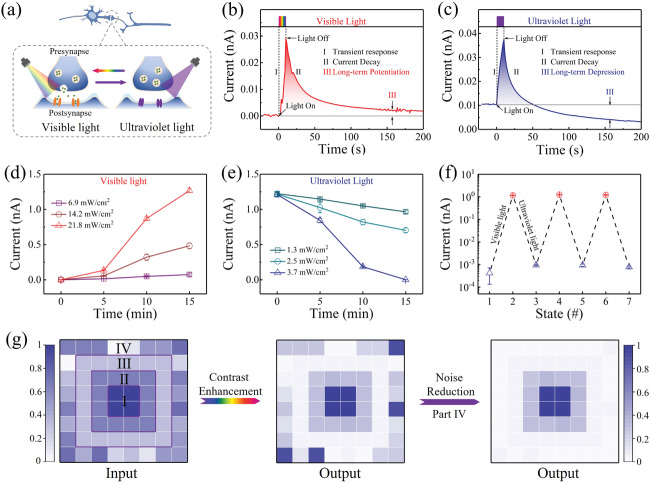
Fully light‐modulated synaptic modification and low‐level image pre‐processing. a) Schematic illustration of the vis light/UV light‐modulated synaptic weights in the artificial synapse. b,c) Synaptic EPSC and IPSC functions when triggered using a vis‐light spike (21.8 mW cm^−2^, 10 s) and a UV spike (3.7 mW cm^−2^, 10 s), respectively. The 0.2 V bias is used to monitor the conductance state of the device. d,e) Light‐induced LTP and LTD as functions of the optical irradiation time (from 0 to 900 s) with various intensities (vis light: 6.9, 14.2, and 21.8 mW cm^−2^; UV: 1.3, 2.5, and 3.7 mW cm^−2^). f) Reversible modulation of the LTP and LTD under alternate stimulations by vis light and UV pulses. g) Low‐level image pre‐processing procedure of our plasmonic optoelectronic memristor, including the contrast enhancement and noise reduction functions. The grey scales in the input image represent different irradiation intensities and those in the output image represent currents. All irradiation intensities and currents are normalized. Left: input image with four components (irradiation intensity I: 1.0; II: 0.65; III: 0.32; IV: random, ranging from 0 to 0.75). Middle: output image after 200 s. Right: reduction of random noise using 300 UV light pulses.

Light‐modulated synaptic modification is first performed to emulate the visual functions in our Au/Ag—TiO_2_/FTO optoelectronic memristor. Here, the device conductance is regarded as the synaptic weight. As illustrated in **Figure** **2**a, the optoelectronic memristor is programmed under both UV light and vis light stimulations, whereas the conductance change *ΔG* is read out through electrical operations (under a bias voltage of 0.2 V). Figure 2b shows the current response of the device under vis light irradiation (21.8 mW cm^−2^, 10 s) within the wavelength region from 400–650 nm (Figure S2, Supporting Information). It is observed that the vis light pulses can induce enhancement of the transient current followed by a decay into a middle state within 200 s, which is similar to the behavior of the excitatory postsynaptic current (EPSC) in a biological synapse.^[^ ^24^ ^]^ Note here that the middle state can be retained for long periods (>1000 s) under dark conditions (Figure S3, Supporting Information). Therefore, the conductance change *ΔG* > 0 is obtained by comparing the conductance values of the initial state and the middle state and indicates that the long‐term potentiation (LTP) of the synaptic weight is induced by vis light stimulation. In contrast, Figure 2c illustrates the current response obtained under UV pulse stimulation (3.7 mW cm^−2^, 10 s) at a wavelength of 350 nm. The UV pulse can trigger a transient current increase; however, the final state after the current decays shows a lower conductance than the initial state, which has relatively high conductance (i.e., *ΔG < 0*). This is similar to the behavior of the inhibitory postsynaptic current (IPSC).^[^ ^44^ ^]^ The low‐conductance state also exhibits long‐term retention characteristics (Figure S3, Supporting Information). This means that the UV pulse is capable of inducing the long‐term depression (LTD) behavior of the synaptic weight in the Ag—TiO_2_ film based synaptic device. In comparison, only the transient photocurrent response can be observed in the pure TiO_2_ film (Figure S4, Supporting Information). Therefore, the results above indicate that both LTP and LTD can be demonstrated reversibly under stimulation by vis light and UV light. This illustrates the full light modulation capability of our optoelectronic memristor, while the similar LTP and LTD behavior are usually performed by hybrid electrical and optical signals in previous optoelectronic memristors.^[^ ^17^, ^25^, ^26^ ^]^ The switching mechanism in this work may be related to oxidation and reduction processes of the Ag nanoparticles under light irradiation,^[^ ^39^, ^40^ ^]^ which will be discussed later in the operating mechanism section. To some extent, the reversible light modulation capability bears a functional resemblance to the light‐stimulated responses of the bipolar cells in biological retina, in which activation and inhibition of the bipolar cells can be tuned reversibly under “light” and “dark” conditions.^[^ ^20^, ^22^ ^]^ Importantly, when compared with the complex hybrid optical/electrical signals that were used in some previous synaptic devices, the fact that the synaptic modification here relies on the optical stimulations alone allows for much simpler signal programming.^[^ ^23^, ^24^, ^25^, ^26^, ^27^ ^]^ The image information with the optical signals can be converted naturally into electrical signals for image sensing.

Complex image sensing and memorization processes usually require responses to the light illumination time and the light density.^[^ ^45^, ^46^, ^47^ ^]^ To examine the above capabilities in our device, we investigated the effects of the light illumination time and the light density on the light‐induced LTP and LTD characteristics, as illustrated in Figure 2d,e, respectively. It is observed that the device conductance increases gradually with increasing vis light illumination time (21.8 mW cm^−2^) until it reaches a saturation state at ≈720 s (Figure S5, Supporting Information), whereas reversible modulation can be implemented using the subsequent UV illumination (3.7 mW cm^−2^). In this work, each conductance point was collected after the light irradiation was paused for 200 s to confirm its long‐term memory feature. Additionally, Figure 2d,e shows the effects of the light density on the LTP and the LTD, respectively. As the light density increased from 6.9 to 21.8 mW cm^−2^, the saturation conductance in the LTP was enhanced from 0.37 to 6.335 nS and the rate of conductance change *ΔG* also increased. Therefore, both the vis light and UV light modulations of our device exhibited time‐dependent and intensity‐dependent characteristics, which allowed more complex image sensing and processing to be performed. Furthermore, Figure 2f shows the results of alternate vis and UV illuminations of the optoelectronic memristor by collecting the conductance values before and after light irradiation (21.8 and 3.7 mW cm^−2^ for the vis and UV illuminations, respectively, 900 s). The results show that the light‐induced LTP and LTD can be operated stably and repeatedly, thus indicating the excellent reproducibility of the device.

Low‐level image pre‐processing is a critical visual capability to optimize the features from a large volume of raw data, which can discard redundant data and compress transferred visual information.^[^ ^27^, ^47^ ^]^ To emulate this low‐level pre‐processing function, the contrast enhancement and noise reduction are demonstrated in an 8 × 8 optoelectronic memristor array, as illustrated in Figure 2g. The training process is used in two steps under vis‐light and UV‐light illuminations. During the vis light training step, the optical image, which is input repeatedly to the memristor array 600 times at a frequency of 1 Hz, contains four regions with different vis light densities (left panel). The densities of regions I, II, and III are set at 21.8, 14.2, and 6.9 mW cm^−2^, which are normalized to values of 1, 0.65, and 0.32, respectively. Note that the optical signals in region IV are designed as the noise input, in which several of the signals have the normalized density of 0 while the others have selected densities ranging randomly from 0.1 to 0.75. The middle panel in Figure 2g shows the image of the output currents (considering the long‐term modifications only) that is obtained after vis light training, in which the normalized currents in regions I, II, and III are 1, 0.3, and 0.04, respectively. Comparison of the input and output images shows that the differences among these three regions are enlarged because of the vis light‐induced LTP function, which indicates the contrast enhancement capacity of our optoelectronic memristor. However, some long‐term noise points are still present in region IV. In previous optoelectronic memristors, only the short‐term noise points could be passively adjusted by the conductance relaxation, while the long‐term noise was not considered.^[^ ^20^ ^]^ Herein, taking the advantage of full light modulation in our memristor, the long‐term noise points can be reduced in an active approach by using UV‐light signals. To demonstrate the noise reduction obtained, the UV light training step is used in region IV with a frequency of 1 Hz (3.7 mW cm^−2^, repeated 300 times). As shown in the right panel of Figure 2g, the noise points are removed because of the specific UV light‐induced LTD function of our optoelectronic memristor, further highlighting the main features in a single image. The results above indicate that our optoelectronic memristor is able to combine the visual sensing function and the low‐level image pre‐processing function.

**Figure 3:**
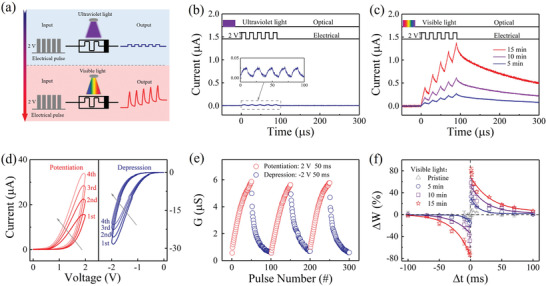
Light‐gated electrically‐driven synaptic plasticity. a) Schematic diagram of the light‐gated electrically‐driven synaptic modification operation. b,c) Current responses under stimulation by pulse strings after UV/vis light irradiation, respectively (2 V, pulse width of 10 µs, interval of 10 µs, read at 0.2 V). d) *I–V* curves illustrating memristive performance of the memristor device after vis light irradiation for 15 min. e) Long‐term conductance potentiation and depression under stimulation by 50 positive/negative pulses (±2 V, 50 ms). f) Synaptic STDP learning rule when modulated using vis light pulses (0, 300, 600, and 900 pulses).

Besides the low‐level pre‐processing (i.e., contrast enhancement and noise reduction), the high‐level image processing with cognitive function is also required in neuromorphic vision for abstract representation of sensory data, for example, recognition, classification, and localization.^[^ ^14^, ^15^, ^16^, ^17^ ^]^ The high‐level image processing is generally performed in artificial neural networks (ANNs) or in spiking neural networks (SNNs).^[^ ^10^, ^19^, ^48^ ^]^ However, the combination of sensing and high‐level image processing functions in a single device is rarely reported. Interestingly, the fully light‐gated synaptic modification, another critical feature of our optoelectronic memristor, provides the possibility to achieve the above functional combination. The light‐gated and electrically‐driven synaptic plasticity, which is an essential ability for pattern recognition in high‐level image processing,^[^ ^47^ ^]^ can also be modulated using a light‐gated terminal in our optoelectronic memristor. As illustrated schematically in **Figure** **3**a, vis light and UV light signals are selected as the gating signals required to activate and inhibit the electrically‐driven synaptic plasticity, respectively. When the memristor device experiences UV illumination (3.7 mW cm^−2^, 15 min) or is in the pristine state (Figure S6, Supporting Information), no obvious current change is observed under stimulation by five positive pulses (2 V, 10 µs), as shown in Figure 3b. This means that the synaptic device remains in a silent state without synaptic plasticity. Interestingly, this silent synapse can be activated through vis light illumination (21.8 mW cm^−2^), in which continuous potentiation of the EPSC can be observed under the same stimulation conditions as before, as illustrated in Figure 3c. These five pulses can also induce the transition from short‐term plasticity to long‐term plasticity, which is a representational phenomenon of the bioactivity of a synapse.^[^ ^49^, ^50^ ^]^ In addition, the silent synaptic device under the higher voltage sweep (±5 V) can only perform abrupt resistive switching between two resistance states rather than the gradual conductance modification (Figure S7, Supporting Information). Furthermore, the learning efficiency of this electrically‐driven synaptic plasticity can be enhanced by increasing the vis light illumination duration from 5 to 15 min. Additionally, the activity of the synaptic device can be tuned reversibly by simply altering the UV light and vis light gating signals.

To present more electrically‐driven synaptic functions, we use the example of a device experiencing vis light illumination for 15 min in the following. Figure 3d shows the current‐voltage (*I–V*) curves for the typical memristive behavior in this device, in which the conductance *G* gradually increases/decreases with consecutive positive/negative simulations. Correspondingly, a series of positive and negative pulses (±2 V, 50 ms) is capable of inducing consecutive potentiation and depression behaviors in our memristive synapse, as illustrated in Figure 3e. In contrast, after UV illumination, the device shows no clear memristive behavior and poor synaptic modification (Figure S8, Supporting Information). Several basic short‐term synaptic functions that are induced using single spikes and paired spikes can be achieved (see Figure S9, Supporting Information), including electrically‐driven EPSC and paired‐pulse facilitation (PPF). As a typical long‐term plasticity, spike‐timing‐dependent plasticity (STDP), which is usually regarded as the essential learning rule for high‐level image processing (pattern recognition) in artificial neural networks,^[^ ^51^, ^52^, ^53^ ^]^ is also demonstrated in the activated synaptic device, as shown in Figure 3f. A pair of pulses (2V/−2V, 50 ms) with the specific interval timing *Δt* that acts as the presynaptic and postsynaptic spikes is applied to the top and bottom electrodes to implement the STDP function. The change in the synaptic weight (*ΔW*) is defined as *ΔW = (G_2_−G_1_)/G_1_*, where *G_1_* and *G_2_* are the long‐term device conductance values measured before and 60 s after application of the paired spikes. As shown in Figure 3f, the paired spikes can induce LTP if the presynaptic spike is earlier than the postsynaptic spike, that is., if *Δt > 0*, whereas the paired spikes result in LTD if *Δt < 0*. In addition, the STDP learning rule can be adjusted further by tuning the illumination time of the vis light gating signal, where longer illumination times can lead to greater conductance changes when using the same electrical pulses. Similarly, the UV illumination can depress the conductance change in the STDP curve by increasing the illumination time (Figure S10, Supporting Information). Thus, the light‐gated modulation of the STDP learning rule may provide a path toward manipulation of the learning efficiency of pattern recognition for high‐level image processing in our optoelectronic memristor, and even its combination with sensing and low‐level pre‐processing functions.

**Figure 4:**
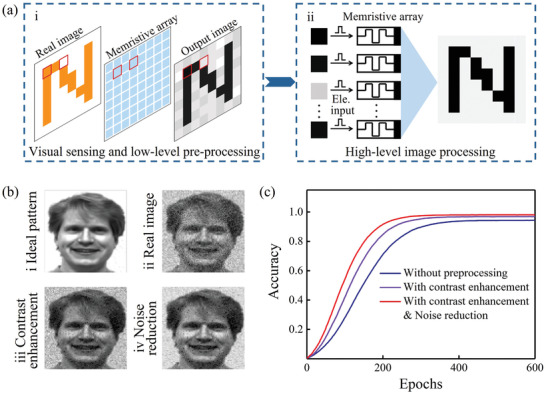
Simulation of functional combination in a neuromorphic visual system with the plasmonic optoelectronic memristor. a) Illustration of the neuromorphic vision system for visual sensing, low‐level image pre‐processing, and high‐level image processing (i.e., recognition) using the plasmonic optoelectronic memristor. b) Images comprising the ideal image (b‐i), the real image with 10% random noise (b‐ii), the contrast‐enhanced image (b‐iii), and the noise‐reduced image (b‐iv) after pre‐processing. Image taken from the Yale Face Database B.^[^ ^61^ ^]^ c) Comparison of image learning and recognition rates with and without the retina‐like pre‐processing, which includes the contrast enhancement and noise reduction functions.

To demonstrate the functional combination of the visual sensing, low‐level image pre‐processing, and high‐level image processing, we constructed a neuromorphic vision system with two components using an 80 × 80 photoelectronic memristor array, as illustrated in **Figure** **4**a. During the first stage (Figure 4a‐i), the real image is first detected and then extracted to realize the sensing and low‐level pre‐processing functions using our optoelectronic memristor, as illustrated in Figure 2. Subsequently, the preprocessed image is transported to a two‐layer artificial neuromorphic network simulator based on our optoelectronic memristor to implement image learning and recognition, as shown in Figure 4a‐ii. Importantly, the memristor array used in these two stages can be the same array in both cases. Here, a grayscale image with 10% noise is selected as the real image for sensing and low‐level pre‐processing. All the light intensity signals shown in the image are normalized. The synaptic weight for each memristor is represented by the gray level of each corresponding pixel. Figure 4b compares the ideal image (Figure 4b‐i) with the real image without processing (Figure 4b‐ii) and the images' output after the image sensing and low‐level pre‐processing stages (Figure 4b‐iii,b‐iv, respectively). The images show that the features of the face image are highlighted because of the contrast enhancement function (Figure 4b‐iii), while the background noise is smoothed further after noise reduction (Figure 4b‐iv). Subsequently, the learning and recognition in high‐level image processing were conducted and compared by inputting the real image without low‐level pre‐processing and the pre‐processed images into the network simulator. The synaptic weight was updated in each learning epoch by following the conductance change *ΔG* of the STDP rule, as illustrated in Figure 3f. Figure 4c shows the results of quantitative analysis of the learning rate and learning accuracy with and without image pre‐processing, in which the learning accuracy is represented by the difference between the ideal image and the updated images. The results show that the recognition accuracy reaches 93% after 400 training epochs without pre‐processing, whereas higher learning accuracy (96%) and a faster learning rate (340 epochs) can be obtained after contrast enhancement. Furthermore, only 300 learning epochs were required to reach a high recognition accuracy of 98% through both contrast enhancement and UV light‐induced background noise reduction. In fact, the vis light‐gated STDP enhancement illustrated in Figure 3f also plays a major role in improving the image learning rate and accuracy. The evolution of these learning processes for the images with and without image pre‐processing is illustrated in Figure S11, Supporting Information. These results indicate that functional combination of visual sensing, the low‐level image pre‐processing (contrast enhancement and noise reduction), and the high‐level image processing (pattern recognition) in our optoelectronic memristor, which may promote the development of efficient artificial visual system.

**Figure 5:**
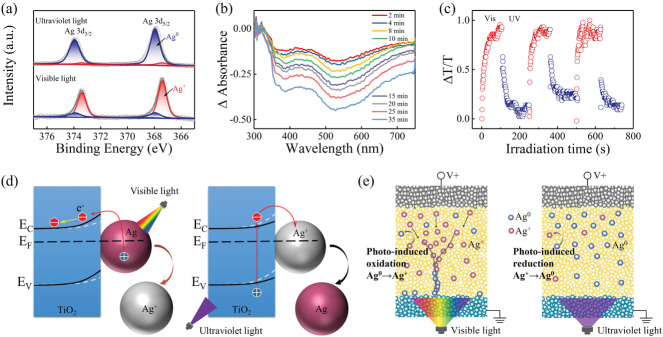
Operating mechanism of the proposed plasmonic optoelectronic memristor. a) XPS spectra of Ag‐doped TiO_2_ nanocomposite after UV and vis light irradiation. b) Evolution of the differential absorption spectra of the Ag‐TiO_2_ film with the green light irradiation time (532 nm, 10 mW per 0.07 cm^2^, from a Nd:YAG laser). c) Relative transmittance change in the Ag—TiO_2_ film under alternating vis light (532 nm, 10 mW per 0.07 cm), and UV irradiation (360 nm, 100 µW per 0.07 cm^2^) cycles. d,e) Schematic diagrams illustrating the mechanisms of light‐induced synaptic modification and electrically‐driven synaptic modification, respectively. Silver atom (Ag^0^) and cation (Ag^+^) are represented by blue and red balls respectively.

We also investigated the fully light‐induced synaptic modification (i.e., conductance change) and light‐gated electrically‐driven synaptic modification mechanisms for our Au/Ag—TiO_2_/FTO plasmonic optoelectronic memristor. To confirm the chemical state of the Ag nanoparticles, X‐ray photoelectron spectroscopy (XPS) characterization of Ag 3d_5/2_ and 3d_3/2_ was performed after UV and vis light irradiation for 15 min, as shown in **Figure** **5**a. Herein, the main peak for 3d^5/2^ could be deconvoluted into two components, in which the peak located at 368/367.4 eV was associated with Ag^0^/Ag^+^. After UV irradiation, the XPS spectrum of the Ag 3d core level confirmed the dominant metallic state of the Ag atoms.^[^ ^54^ ^]^ In addition, the vis light irradiation induced a clear shift toward the lower binding energy value for Ag 3d, which indicates the increase of Ag^+^ according to the literature.^[^ ^55^ ^]^ Therefore, from analysis of the XPS spectra, the light‐induced conductance change was likely to be related to photo‐induced oxidation and reduction of the Ag nanoparticles, which is similar to the photochromic mechanism of Ag—TiO_2_ described in the literature.^[^ ^36^, ^56^ ^]^ Furthermore, as illustrated in Figure 5b, the differential absorption spectrum of the Ag—TiO_2_ film was used with increasing vis light (532 nm) irradiation times to investigate the LSPR effect of the Ag nanoparticles. As indicated in Figure 1g, the presence of absorption bands in the visible region (≈500 nm) can be attributed to the LSPR absorption behavior of the Ag nanoparticles. Here, it is observed that the absorption intensity clearly decreases with increasing vis light irradiation time, which may be the result of morphology changes in the Ag nanoparticles induced by Ag oxidation.^[^ ^37^, ^38^, ^39^ ^]^ Additionally, the light‐induced synaptic modification can be also monitored using an optical reading approach, which uses the transmittance of a probe beam (532 nm, 10 µW per 0.07 cm^2^) through the Ag—TiO_2_ film (Figure S12, Supporting Information). As illustrated in Figure 5c, the relative transmittance change (*ΔT/T*, where the pristine film is used as the reference) can be tuned reversibly under UV (360 nm, 100 µW per 0.07 cm^2^) and vis light irradiation (532 nm, 10 mW per 0.07 cm^2^), indicating the potential of the structure for application not only to optoelectronic memristors but also to long‐term holographic data storage.^[^ ^40^, ^56^ ^]^

The experimental analyses described above demonstrate that the oxidation/reduction processes of the Ag nanoparticles play a major role in the operating mechanism of our plasmonic optoelectronic memristor. A general model is therefore proposed to explain the light‐modulated synaptic modification process, as illustrated in Figure 5d. Under vis light irradiation, hot electrons are excited through the LSPR effect of the Ag nanoparticles and are then transferred to the conductance band of the TiO_2_ because of the existence of a Schottky barrier at the micro–nano interface of the Ag/TiO_2_ material.^[^ ^57^ ^]^ Importantly, the charge separation between the excited electrons and the Ag^+^ enables photo‐oxidation of the Ag nanoparticles (Ag^0^ → Ag^+^ + e^−^) and reduces the Schottky barrier at the Ag/TiO_2_ interface, thus resulting in promotion of the electronic conductivity (i.e., vis light‐induced LTP). Conversely, UV irradiation can excite the electrons from the valence band to the conduction band of TiO_2_, which can then lead to recombination of the electrons with Ag (Ag^+^ + e^−^ → Ag^0^), that is, photo‐reduction of Ag^+^. The possible electron transport processes in the Ag—TiO_2_ film under the vis light and UV light are discussed in Figure S13, Supporting Information. For the mechanism of the electrically‐driven synaptic modification, we generally attributed the origin of multiple states to the change of effective diameter of Ag conducting filaments (CFs) driven by the consecutive voltage pulses, through the electrochemical metallization (ECM) redox process.^[^ ^58^, ^59^, ^60^ ^]^ The conventional ECM process usually includes the following steps (Figure S14, Supporting Information): (i) electrically‐driven Ag oxidation, that is, Ag^0^→ Ag^+^; (ii) Ag^+^ migration through the switching layer; and (iii) Ag^+^ reduction at the cathode (Ag^+^→ Ag^0^), which results in the growth and modulation of successive Ag clusters (i.e., Ag CFs). Importantly, the step (i) of Ag oxidation may be avoided by using vis light illumination in current work, because a large number of Ag^+^ ions can be generated and adjusted through the vis light illumination, as illustrated in Figure 5e. The skip of step (i) can effectively reduce the operation voltage for memristive switching. Therefore, the electrical pulses with relatively small bias (i.e., 2 V, 10 µs) can migrate these Ag ions through the whole film and gradually modulate the effective diameter of Ag CFs for multiple conductance states (Figure 3c). In contrast, the quantity of Ag atoms in film increases with UV light illumination (Figure 5e), in which the step (i) of Ag oxidation driven by high voltage should be required for conductance switching. This may be the reason that the relatively weak pulses (2 V, 10 µs) are unable to induce clear memristive switching behavior in Figure 3b. Furthermore, the quantity of Ag^+^ and Ag^0^ in step (i) can be tuned by the irradiation time and intensities of vis light and UV light, thus accounting for the light‐gated electrically‐driven synaptic modification process illustrated in Figure 3f.

## 3. Conclusion

In this work, we have demonstrated a plasmonic optoelectronic memristor for the first time that relies on a combination of the LSPR effect and optical excitation in Ag—TiO_2_ nanocomposite. The proposed device can perform fully light‐induced synaptic modification and light‐gated electrically‐driven conductance change processes, which enable the combination of sensing, low‐level image pre‐processing, and high‐level image processing functions to be performed in a single device. Several synaptic functions have been demonstrated in this device, including short‐term/long‐term plasticity and STDP learning rule. In particular, the electrically‐driven STDP rule can be modulated by using the light as the gating terminal. By performing the low‐level image pre‐processing of the contrast enhancement and noise reduction steps, the learning rate and efficiency of the high‐level image recognition process can be improved effectively in our memristors, as indicated by simulations. We believe that our study offers a new type of plasmonic optoelectronic memristor and paves the way toward application of these memristors to provide highly efficient neuromorphic vision.

## 3 Experimental Section

### Device Fabrication

Memristors with Au/Ag—TiO_2_/FTO sandwich structures were fabricated on FTO substrates and then patterned into memristive arrays with areas of 500 × 500 µm^2^ using a metal mask. First, the TiO_2_ film was fabricated using a dip‐coating method from an equivolume mixture solution composed of TiO_2_ nanoparticles (STS‐01, 0.4 mol L^−1^, Ishihara Sangyo) and PEO20‐PPO70‐PPO20 block copolymer (20 g L^−1^). The nanoporous structure was obtained by annealing at 450 ℃ to remove the polymer. The Ag–TiO_2_ nanocomposite film was then fabricated by immersing the structure in an AgNO_3_ solution (0.1 mol L^−1^) and irradiating it using UV light (360 nm, 100 µW cm^−2^) for 15 min. Finally, the Au electrodes (500 µm) were deposited on the top of the film to act as electrodes by sputtering.

### Measurement and Characterization

The electrical measurements were performed using instruments that include arbitrary function generators (3390, Keithley; TGA12104, TTI), an oscilloscope (DSOS404A, Keysight), and a sourcemeter (2636A, Keithley). The optical modulation during the electrical measurements was performed using a xenon lamp (LA‐410UV, Hayashi).

### Optical Setup

During differential absorption and transmitted intensity measurements, visible light (532 nm, 10 mW per 0.07 cm^2^) was selected as the pump light, UV light (360 nm, 100 µW per 0.07 cm^2^) was selected as the erasing light, and ultra‐low intensity visible light (532 nm, 10 µW per 0.07 cm^2^) was used as the probe light.

### Statistical Analysis

The device conductance was calculated by the equation: *G* = *I*/*V*. The current and voltage were recorded directly with an oscilloscope and sourcemeter. For the normalization of irradiation intensities and currents, the strongest and weakest signals corresponded to 1 and 0, respectively. The Origin software was used for data processing and analysis.

## Conflict of Interest

The authors declare no conflict of interest.

## Supporting information

Supporting Information

## Data Availability

The data that support the findings of this study are available from the corresponding author upon reasonable request.
