# Supplementary material for: Plasmonic Optoelectronic Memristor Enabling Fully Light‐Modulated Synaptic Plasticity for Neuromorphic Vision
Source: Adv Sci (Weinh). 2021 Dec 29;9(6):2104632. doi: 10.1002/advs.202104632 (PMC8867191; doi:10.1002/advs.202104632)
Supplement: Supplementary file 1 — Supporting Information [file ADVS-9-2104632-s001.pdf]

ADVANCED SCIENCE
Open Access

Supporting Information

for *Adv. Sci.,* DOI: 10.1002/advs.202104632

Plasmonic optoelectronic memristor enabling fully light-modulated synaptic plasticity for neuromorphic vision

Xuanyu Shan, Chenyi Zhao, Xinnong Wang, Zhongqiang Wang, Shencheng Fu, Ya Lin, Tao Zeng, Xiaoning Zhao, Haiyang Xu*, Xintong Zhang and Yichun Liu**

Copyright WILEY-VCH Verlag GmbH & Co. KGaA, 69469 Weinheim, Germany, 2016.

Supporting Information

Title: Plasmonic optoelectronic memristor enabling fully light-modulated synaptic plasticity for neuromorphic vision

Xuanyu Shan, Chenyi Zhao, Xinnong Wang, Zhongqiang Wang*, Shencheng Fu, Ya Lin, Tao Zeng, Xiaoning Zhao, Haiyang Xu*, Xintong Zhang and Yichun Liu*

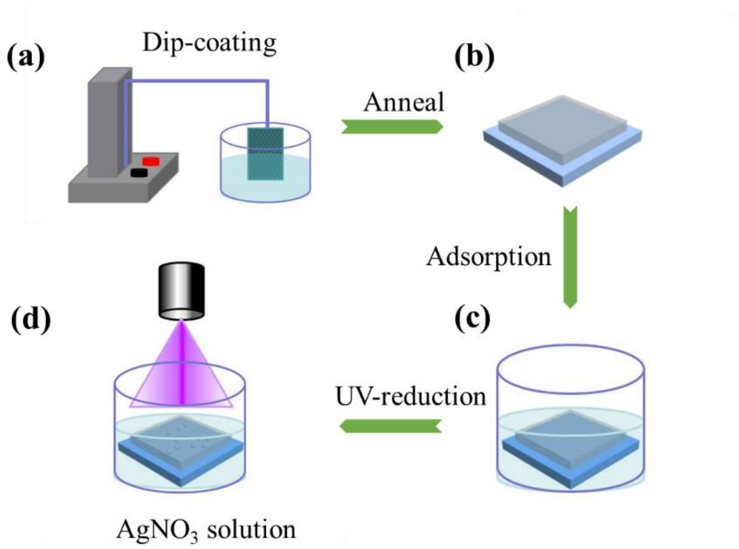

Figure S1. Schematic diagram of the fabrication of Ag-TiO_2 nanocomposite film. (a) TiO_2 film is prepared on FTO substrate using the dip-coating technique. (b) Removing the polymer by heat treatment. (c) Immersing the TiO_2 nanoporous film into the $AgNO_3$ solution. (d) Depositing Ag nanoparticles with ultraviolet light. The Ag-TiO_2 nanocomposite film is synthesized based on the procedures as reported in our previous literatures.

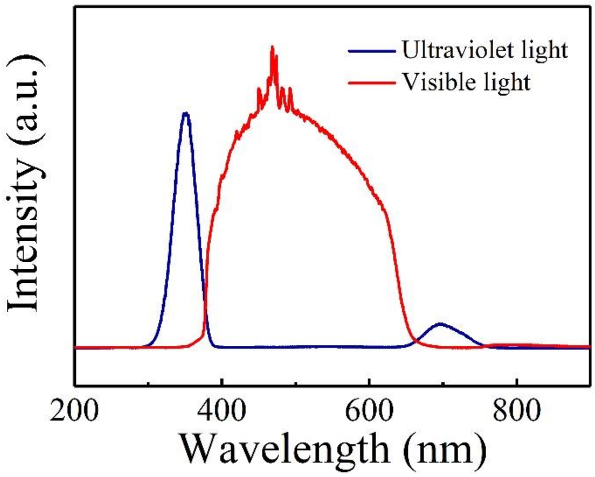

Figure S2. The spectrum distribution of ultraviolet light and visible light source. Herein, the ultraviolet light and visible light is obtained with color filter and xenon lamp (LA-410UV). The visible light exhibites a wavelength region of 400-650 nm and the ultraviolet light has the spectra centered at 350 nm.

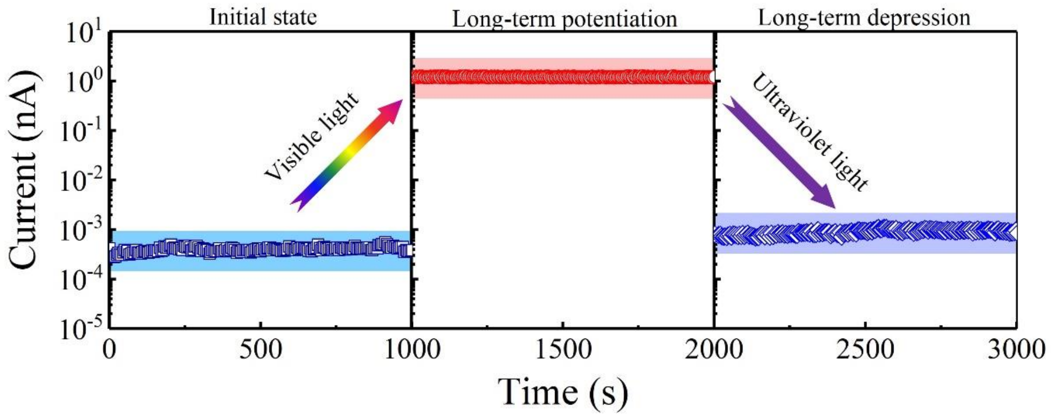

Figure S3. Long-term potentiation/depression induced by visible/ultraviolet light. After the Vis-light irradiation (21.8 mW/cm^2, 900 s), the device exhibits long-term potentiation behavior, which remains stable for more than 1000 s. On the other hand, device conductance exhibits long-term depression after ultraviolet irradiation (3.7 mW/cm^2, 900s). All the device conductance is monitored with the bias of 0.2 V under dark conditions.

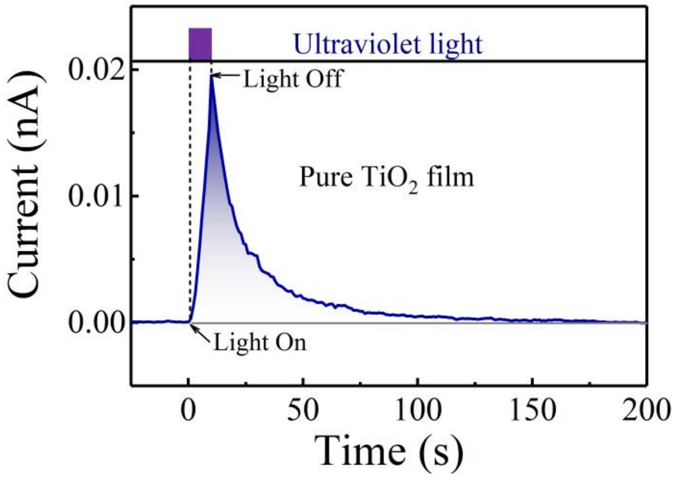

Figure S4. Current response of pure TiO_2 film under ultraviolet light illumination.

We have measured the current response of pure TiO_2 film induced by ultraviolet light, as shown in Figure S4. It can be observed that only a transient increase of photocurrent can be triggered by the UV light, which entirely decays to the initial state after ~130 s. This transient photocurrent is essentially different from the long-term depression behavior in Ag-TiO_2 nanocomposite film as seen in Figure 2c. The above results indicate that the UV light induced long-term depression in our optoelectronic memristor cannot be attributed to the photoconduction of TiO_2 film.

One may doubt if the long-term conductance potentiation is induced by the trapped electrons. This possibility can be excluded by measuring the photocurrent of pure TiO_2. If the trapped electrons are responsible, the long-term conductance change should be observed in this experiment. However, it is seen that only a transient increase of photocurrent can be triggered by the UV light, in which the trapped electrons are entirely released and the current decays to the initial state after ~130 s. Thus, the long-term conductance change origins from the oxidation and reduction of Ag atoms in our work, rather than the trapped electrons.

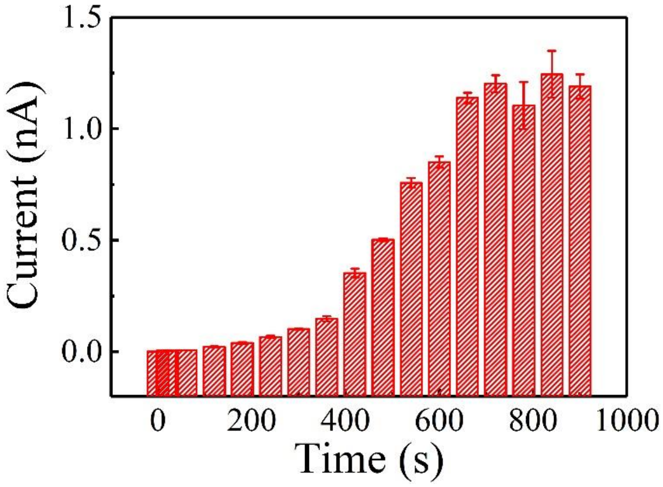

Figure S5. The light-induced LTP as the function of visible irradiation time (21.8 mW/cm^2). The device conductance exhibits strong irradiation-time dependence. The initial current increase significantly with irradiation time increasing to 720 s and then reaches the saturation state. Herein, we demonstrate the light-induced LTP with visible light as irradiation source.

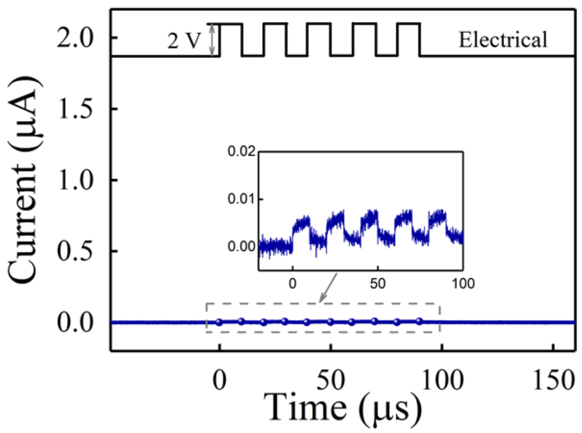

Figure S6. The current response of pristine device. Pristine device remains in a high resistance state without exhibiting obvious current change, which means pristine synaptic device is in silent state. The spike train consists of 5 positive pulse (2V, pulse width 10 μs, interval 10 μs, read at 0.2 V).

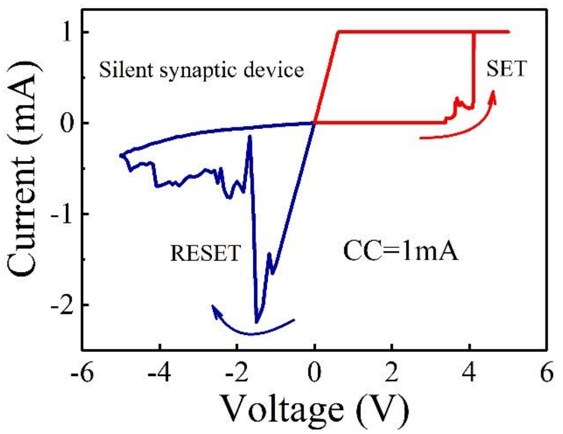

Figure S7. I-V curve of silent synaptic device under the higher voltage sweep (±5 V).

The I-V curve of silent synaptic device under the higher voltage sweep (±5 V) was measured, as shown in Figure S7. It is seen that the abrupt resistive switching (RS) occurs with a SET voltage of ~3.4 V and RESET voltage of ~-1.8 V, which is generally called as digital resistive switching (D-RS). Although the transition between high-resistance-state (HRS) and low-resistance-state (LRS) can be obtained in such D-RS behavior, it cannot be regarded as the activation of synaptic device. Because the synaptic device usually needs multiple resistance states or conductance states to demonstrate the synaptic plasticity function.

As a clear comparison, the device activated by Vis light illumination can present gradual conductance modification using consecutive positive/negative pluses (±2 V, 50 ms) with a relatively small bias, as shown in Figure 3d and 3e. The above results mean that the activation of Vis light illumination cannot be replaced by a higher electrical voltage in our study of synaptic emulation.

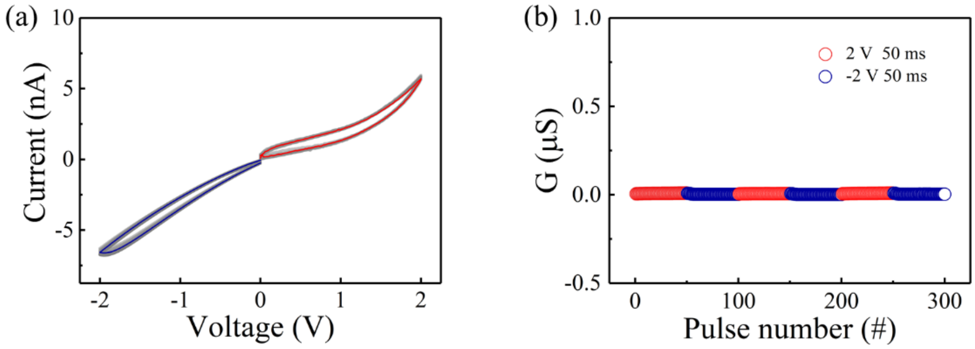

Figure S8. Memristive behavior of UV-irradiated device. (a) I-V curves of Ag-TiO_2 device after UV-light illumination (15 min), showing no memristive switching. (b) device conductance under the repeated stimulation of 50 positive/negative pulse (±2V, 50 ms). There is no conductance potentiation and depression under the stimulation.

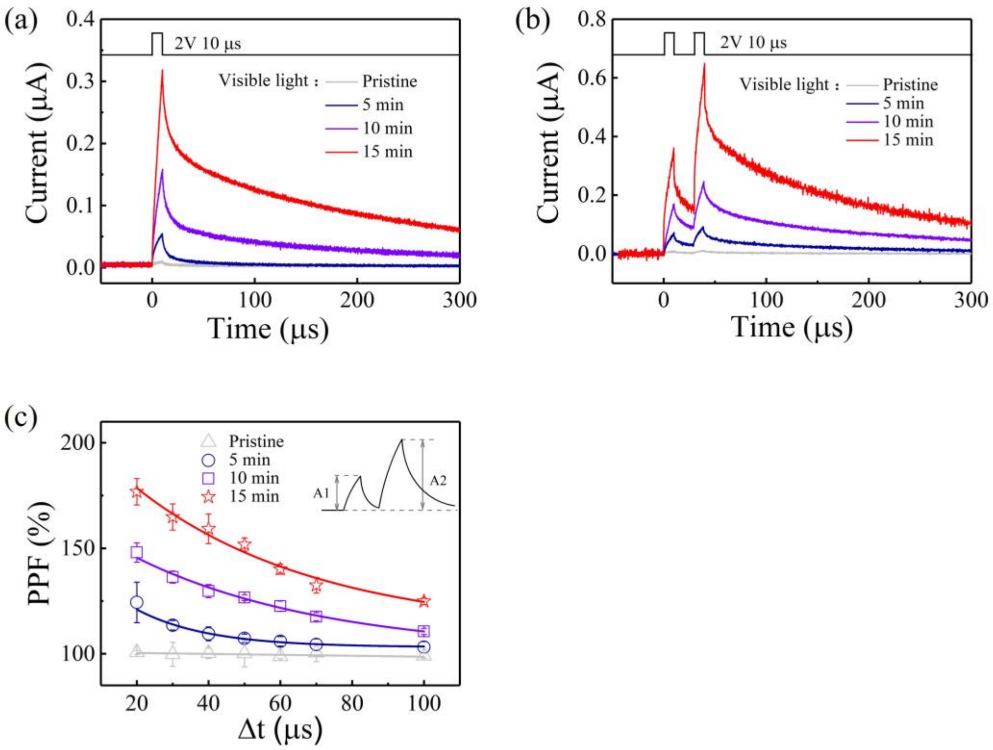

Figure S9. Short-term synaptic functions modulated with Vis-light illumination time. (a) synaptic EPSC triggered by electrical pulse (2 V, 10 μs) after the Vis-light illumination (0, 5, 10, 15 min respectively). As Vis-light illumination time increases, the electrical pulse enables greater increases and slower decay in current.

(b) synaptic functions of paired-pulse facilitation (PPF).

(c) PPF index dependent on relative spike timing. The PPF can be calculated with

$$\mathrm{PPF} = 100\% \times \frac{\mathrm{A_2}}{\mathrm{A_1}}$$

Shorter interval time between two spikes induces greater synaptic facilitation, while Vis-light illumination can enhances the correlation.

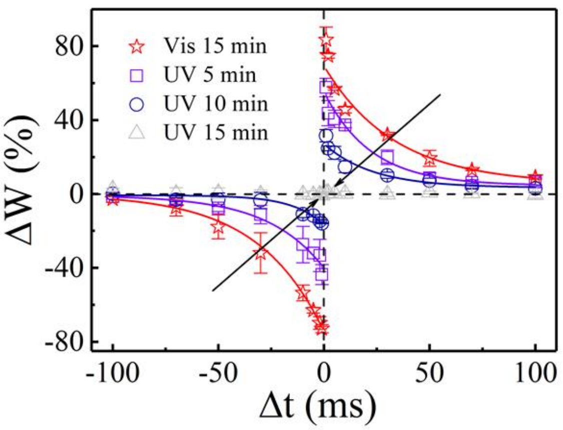

Figure S10. Synaptic STDP learning rule modulated with various UV-light illumination (5, 10, 15 minutes respectively). Under the action of same electrical signals, UV-light illumination can decrease the change of synaptic weight (*ΔW*). Before the ultraviolet irradiation, the device is irradiated with visible light for 15 minutes.

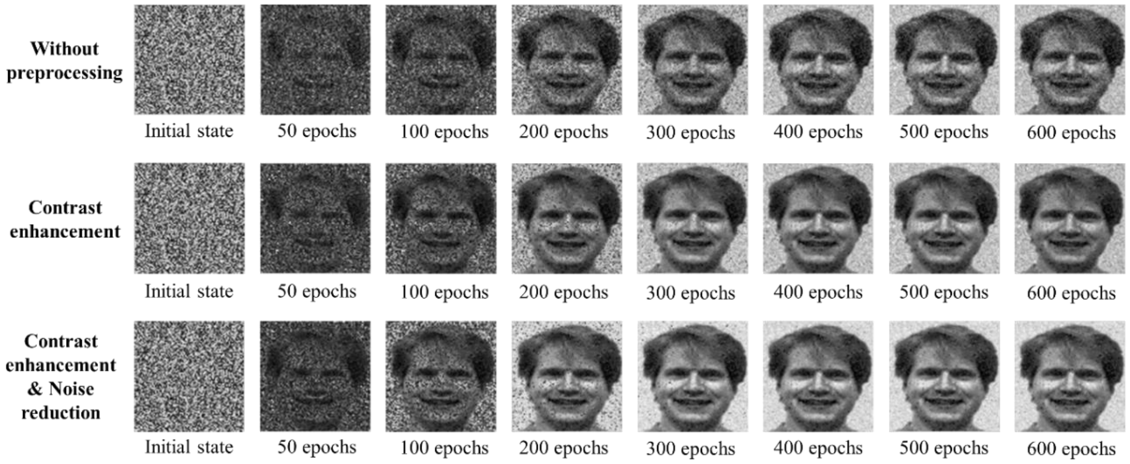

Figure S11. The image evolution during learning process (with/without pre-processing) of artificial neuromorphic network (ANN). Herein, the learning processes followed the conductance change ΔG of STDP rule. The ANN with low-level pre-processing (contrast enhancement and noise reduction) possessed better learning speed and accuracy. The quantitative analysis was demonstrated in figure 4c.

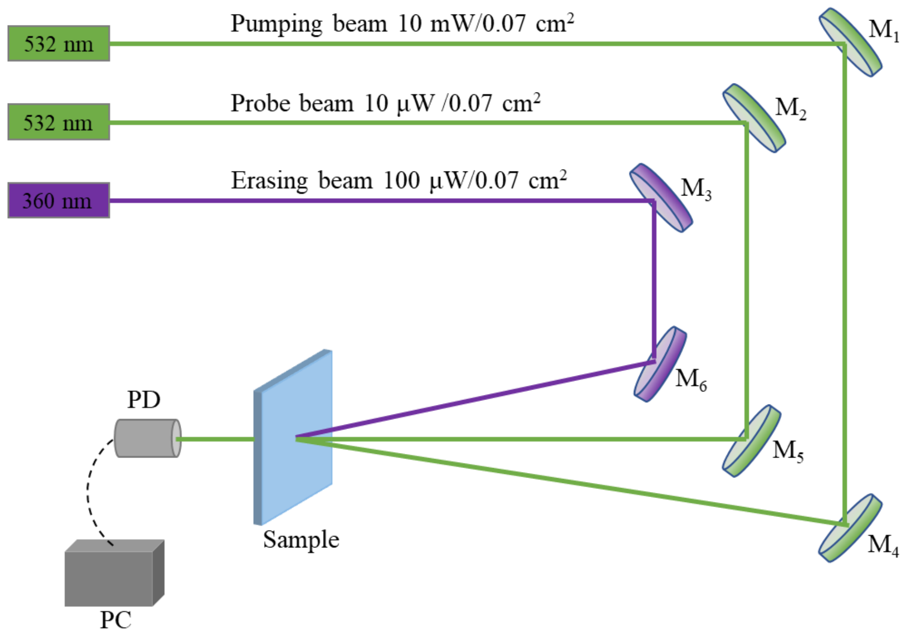

Figure S12. Optical setup for photoinduced transmittance variation in Ag-TiO_2 film. (M, mirror; PD, photodiode). Pumping beam (532 nm, 10 mW/0.07 cm^2) and erasing beam (360 nm, 100 μW/0.07 cm^2) were used to modulate the trasmittance of Ag-TiO_2 film. The probe beam (532 nm) was set to 10 μW/0.07 cm^2 to minimize the destructive effect. The transmittance variation is obtained by comparing the probe beam intensity detected by PD. With the brilliant laser directionality, pumping beam and erasing beam can be adjusted at different angles to minimize the impact on probe beam. The optical setup refers to the our previous literatures.

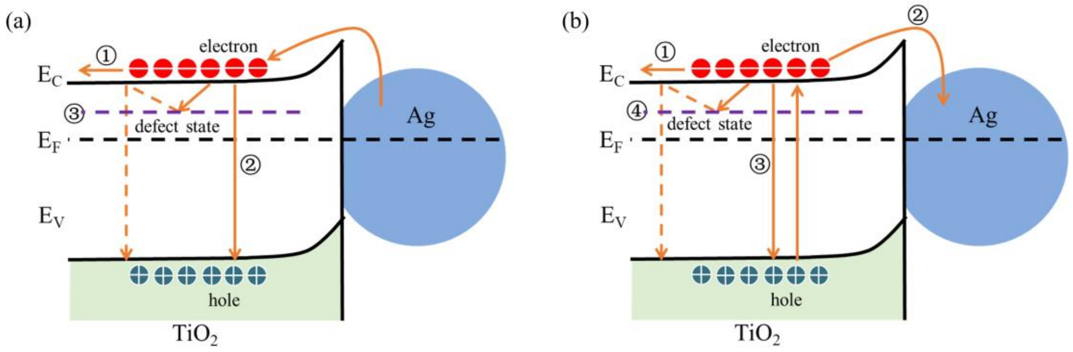

Figure S13. Schematic diagram of electron transport in the Ag-TiO_2 film under Vis light and UV light.

The Vis light illumination can excite the electrons from Ag atom to the conduction band of TiO_2 film by LSPR effect, which leads to excess carrier and increases the transient photocurrent (①), as shown in Figure 2b and Figure S13a. During the recovery process after Vis light illumination, part of the photogenerated electrons immediately combine with holes (②), while part of them are trapped by the defect states in the film (③). Then, the trapped electrons may be released to the conductance band later and eventually combine with holes, which results in the slow photocurrent decay. Herein, the excitation of electrons from Ag atoms also results in the Ag oxidation and long-term conductance potentiation.

The UV light illumination can excite the electrons from the valence band to conduction band, in which the excess carrier certainly can increase the transient photocurrent (①) as shown in Figure 2c and Figure S13b. At the same time, part of photogenerated electrons can be transfer to the Ag ions for reduction (②), resulting in a long-term change of Ag atoms. This process can be supported by the experimental data of Figure 5 (a). During the recovery process after UV light illumination, part of the photogenerated electrons immediately recombine with holes (③), while part of them are trapped by the defect states in the film (④). The trapped electrons may be released to the conductance band later and eventually recombine with holes, which results in the slow photocurrent decay for 200s as shown in Figure 2c. Therefore, process ④ still can generate excess carrier for high transient current

for the first 50s. In addition, long-term conductance depression is indeed produced by the process ②, which can be only monitored after the photocurrent decay.

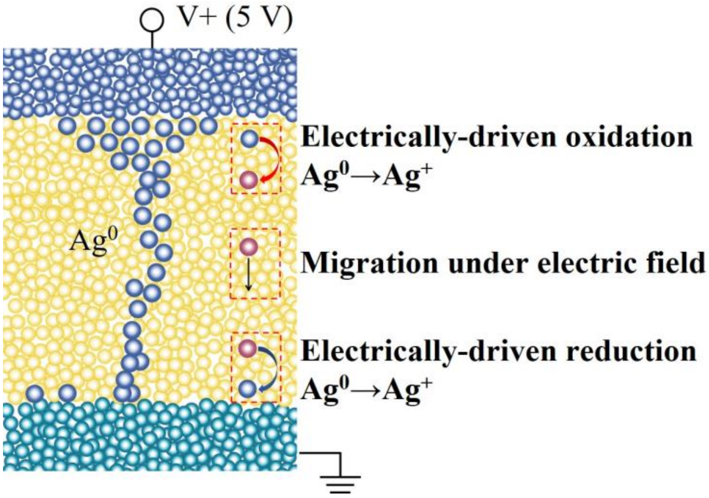

Figure S14. Conventional ECM process i) electrically-driven Ag oxidation (ii) Ag^+ migration through the switching layer; and (iii) Ag^+ reduction at the cathode.
